# Comparative Efficacy of Postoperative Pain Management Techniques Following Costal Cartilage Harvest: A Systematic Review and Network Meta-analysis

**DOI:** 10.1007/s00266-024-04430-2

**Published:** 2024-11-11

**Authors:** Jihan Guo, Xin Li, Jie Li, Xiaoning Yang, Lu Yu, Tailing Wang

**Affiliations:** Department of Facial and Cervical Surgery, Plastic Surgery Hospital, Chinese Academy of Medical Sciences and Peking Union Medical College, No.33, Ba Da Chu Road, Shi Jing Shan District, Beijing, 100144 China

**Keywords:** Costal cartilage harvest, Pain management, Multimodal analgesia, Network meta-analysis, Postoperative pain, Adverse effects

## Abstract

**Background:**

Efficient pain control is essential in reconstructive surgeries, particularly in procedures involving the harvest of costal cartilage. This study examines and compares different pain relief treatments using a network meta-analysis (NMA) to determine the most effective techniques for managing pain.

**Methods:**

We performed a systematic review and network meta-analysis (NMA) by scanning several databases such as PubMed, Embase, Cochrane Central Register of Controlled Trials (CENTRAL), China National Knowledge Infrastructure (CNKI), Wanfang, and Weipu till March 18, 2024. The review analyzed randomized controlled trials and observational studies that evaluated the effectiveness of local anesthetics and multimodal analgesia techniques in treating postoperative pain following costal cartilage harvest. Primary outcomes were pain scores at 6-, 12-, 24-, and 48-h post-surgery, while secondary outcomes included the need for rescue analgesia and opioid-related adverse effects.

**Results:**

Fourteen studies involving 935 participants were included. The analysis revealed that multimodal strategies, particularly ‘Methylene Blue and Ropivacaine Intercostal Nerve Block (MB & Ropivacaine ICNB) combined with Patient-Controlled Analgesia (PCA),’ were the most effective techniques to reduce pain scores across 6, 24, and 48-h time points. “Pre-operation SAPB & PSB + PCA” was most effective in reducing pain score at 12h and significantly decreased the need for rescue analgesia and opioid-related adverse effects. In contrast, traditional ICNB with single drug consistently showed the least efficacy.

**Conclusion:**

The results of our study strongly support the use of multimodal analgesic techniques instead of typical single medication ICNB for managing postoperative pain after costal cartilage harvest. These strategies not only provide superior pain control but also contribute to reducing the dependency on opioids, aligning with current clinical priorities to enhance recovery and minimize opioid-related risks.

**No Level Assigned:**

This journal requires that authors assign a level of evidence to each submission to which Evidence-Based Medicine rankings are applicable. This excludes Review Articles, Book Reviews, and manuscripts that concern Basic Science, Animal Studies, Cadaver Studies, and Experimental Studies. For a full description of these Evidence-Based Medicine ratings, please refer to the Table of Contents or the online Instructions to Authors www.springer.com/00266.

**Supplementary Information:**

The online version contains supplementary material available at 10.1007/s00266-024-04430-2.

## Introduction

Costal cartilage is a pivotal autologous material and is commonly employed in reconstructive surgeries. It is particularly utilized in procedures like nose reconstruction, microtia repair, and maxillofacial reconstruction [1, 2]. Although the harvest of costal cartilage has advantages, it often causes substantial pain at the donor site [3], which can hinder postoperative recovery by limiting coughing and early movement. This pain may lead to complications such as atelectasis, ventilation/perfusion mismatch, hypoxemia, and potential infections [4]. Effective management of postoperative pain is therefore critical, as it enhances respiratory function, prevents chronic pain, facilitates early mobility, and improves overall patient compliance and satisfaction [5].

Conventional approaches that include administering pain-relieving drugs through injections or orally frequently have limited effectiveness and can cause significant systemic side effects, such as respiratory suppression. This is particularly concerning when treating children [6, 7]. Consequently, there has been a shift toward exploring advanced pain management techniques. These include but are not limited to intercostal nerve blocks (ICNB), which involve the injection of local anesthetics and other medications into the intercostal spaces to block pain signal transmission. Commonly used analgesics for ICNB include Ropivacaine, Methylene Blue (MB) [8–10], Betamethasone, and Triamcinolone Acetonide, or combinations of these drugs [11–13]; catheter-based continuous wound infiltration (CWI) [14]; and ultrasound-guided blocks such as the serratus anterior plane block (SAPB) and paravertebral nerve block (PVB) [15–17]. Additionally, innovations like the improved parasternal block (PSB) [18] and strategic variations in the timing and combination of analgesic methods have also been introduced [19].

Although there are many other ways available, much of the present research primarily focuses on comparing these innovative approaches to classic procedures like single drug ICNB with patient-controlled analgesia (PCA) [20, 21]. A comparative analysis of these newer techniques against one another, however, remains notably absent from current literature. To address this gap, we have undertaken a systematic review and network meta-analysis. This study’s objective is to compare the efficacy of different advanced pain management techniques in reducing postoperative pain and to evaluate opioid-related adverse effects. The goal is to provide plastic surgeons and their patients with evidence-based suggestions for improving postoperative results in procedures involving the harvest of costal cartilage.

## Methods

This systematic review and network meta-analysis were registered in the PROSPERO database (Registration No. CRD42024534222) prior to conducting the review. The study was conducted in strict adherence to the PRISMA (Preferred Reporting Items for Systematic Reviews and Meta-Analyses) guidelines [22] to ensure methodological rigor and transparency.

### Search Strategy

A comprehensive literature search was conducted across several databases, including PubMed, Embase, Cochrane Central Register of Controlled Trials (CENTRAL), China National Knowledge Infrastructure (CNKI), Wanfang, and Weipu. The search included the entire database history up until March 18, 2024, and did not impose any language limitations. The search aimed to identify studies assessing the efficacy of postoperative pain management in costal cartilage harvest. The specific keywords used in the search included “rhinoplasty”, “congenital microtia”, “ maxillofacial reconstruction”, “costal cartilage”, “anesthesia”, “analgesia”, “postoperative pain control”, “ donor site analgesia”.

The precise search strategy for each database is provided as a supplementary file, detailing the combination of keywords and Boolean operators used. To ensure comprehensiveness, reference lists of relevant articles and reviews were also examined for additional studies. Two researchers (GJH and LX) independently screened titles and abstracts to identify potentially relevant studies. Following this, the same two researchers independently performed full-text screening to determine the final inclusion of studies. Any disagreements between the two researchers were resolved through discussion. If consensus could not be reached, a third author (WTL) was consulted to make the final decision.

### Study Selection

After conducting a thorough search across various databases, the detected records were carefully selected to ensure they were relevant and aligned with the aims of the study. Initially, duplicates were removed, streamlining the pool of potential studies for inclusion. Subsequently, two independent reviewers (LJ and YL) conducted a preliminary screening based on titles and abstracts, filtering out studies that clearly did not meet the inclusion criteria. The remaining studies underwent a thorough examination of the full text by the two reviewers independently. Any conflicts among reviewers were handled through discourse or, if required, by seeking input from a third adjudicating author (WTL).

The inclusion and exclusion criteria were defined using the PICOS format:

1. Population (P):
  Inclusion: Patients undergoing costal cartilage harvest for reconstructive surgery, including rhinoplasty, microtia repair, and maxillofacial reconstruction.
  Exclusion: Studies focusing on patients with other types of surgeries or those involving non-autologous rib grafts.
2. Intervention (I):
  Inclusion: Various advanced pain management techniques, including intercostal nerve blocks (ICNB) with local anesthetics, multimodal analgesia, catheter-based continuous wound infiltration (CWI), and ultrasound-guided nerve blocks (e.g., serratus anterior plane block, paravertebral nerve block).
  Exclusion: Studies that did not focus on pain management strategies or used systemic analgesics without local interventions.
3. Comparator (C):
  Inclusion: Comparisons between different pain management techniques, including single-agent ICNB, multimodal approaches, and other advanced analgesic strategies.
  Exclusion: Studies without a comparator group or those comparing non-relevant interventions.
4. Outcomes (O):
  Inclusion: Primary outcomes included pain scores at 6, 12, 24, and 48 h post-surgery. Secondary outcomes included the need for rescue analgesia and the incidence of opioid-related adverse effects.
  Exclusion: Studies that did not report on these outcomes or lacked extractable data.
5. Study Design (S):
  Inclusion: Randomized controlled trials (RCTs) and observational studies.
  Exclusion: Case reports, reviews, animal studies, and non-clinical research.

### Data Extraction

After being retrieved, all pertinent articles found in the specified databases were organized in EndNote X9 for systematic administration. Two writers (GJH and YXN) independently performed data extraction from the studies that matched the inclusion requirements. Any differences were handled through consensus among all contributing authors. The extracted data encompassed key publication details (author names, title, publication year, and journal), patient demographics (age and sex), details of the interventions (type of intervention, timing, and effectiveness of both the intervention and placebo), and clinical outcomes.

When extracting data, if a study included standard errors for both the experimental and control groups, we calculated the standard deviations using the formula: SD = SE×√*n*. If standard deviations or standard errors were not provided, the estimation of standard deviations was done using confidence intervals, *t*-values, quartiles, ranges, or *p*-values as outlined in section 7.7.3 of the Cochrane Handbook for Systematic Reviews [23]. If critical data could not be obtained through these methods, efforts were made to contact the authors of the study up to four times over a six-week period to request the necessary information.

### Outcomes

The main objective of this study is to measure the level of pain experienced by patients after surgery at specific time intervals (6, 12, 24, and 48 h) while at rest and during coughing. The pain will be evaluated using well-established and validated scales: Visual Analog Scale (VAS) [24], FLACC Scale [25], Numeric Rating Scale (NRS) [26], and Facial Expression Scale [27]. These scales assign scores ranging from 0 to 10, with lower scores indicating lower levels of pain. Secondary outcomes encompass the rate at which rescue analgesics are used and the occurrence of adverse effects related to opioids, such as nausea, vomiting, respiratory depression, constipation, and pruritus.

### Risk of Bias

Two authors (LX and LJ) independently evaluated all included RCT studies using the Cochrane Risk of Bias Tool (RoB-1) [28], following the systematic review’s principle of inclusion by not removing studies based on their bias appraisal outcomes. The assessment produced a summary of bias risk and a graph using RevMan version 5.4, which provided a clear and comprehensive visual representation of the bias risk in different research. The quality of each RCT study was meticulously assessed following the methodology outlined in the Cochrane Handbook version 5.1.0, which encompasses seven key areas: randomization method, allocation concealment, blinding of participants and personnel, blinding of outcome assessment, completeness of outcome data, selective outcome reporting, and other potential sources of bias. Each domain’s risk of bias was categorized as low, high, or unclear, facilitating a nuanced grading of study quality.

The Newcastle-Ottawa Scale (NOS) [29] was used to assess the quality of non-randomized intervention studies, namely cohort studies. This approach underscores the rigorous evaluation of methodological quality and bias across study designs. The analysis of outcomes adopted an intention-to-treat principle with a particular focus on the treatment of missing data. A worst-case scenario analysis was applied to dichotomous outcomes, assuming non-response in participants with missing information. Studies that exhibited outcomes that were reversed under this situation were deemed to possess a significant risk of attrition bias. Moreover, if the potential impact of missing outcomes on the observed effect size is substantial, the study was considered to have a considerable risk of attrition bias.

### Data Synthesis

Statistical analysis and data synthesis were conducted using STATA software version 15.0 utilizing specialized packages including mvmeta, network, st0411, and sencode to facilitate comprehensive meta-analyses. Sensitivity analysis was performed to assess the robustness of the findings employing the same software suite. The level of heterogeneity among the studies was assessed using STATA to calculate the *I*^2^ statistic and chi-square tests, considering *P* < .1 and *I*^2^ > 50% as significant heterogeneity thresholds. Additionally, the publication bias for primary outcomes and adverse events was evaluated by inspecting the funnel plot.

Given the different pain assessment tools used in the included studies, we used the Standardized Mean Difference (SMD) to synthesize continuous variables, ensuring comparability across different scales. Relative risks (RRs) and 95% confidence intervals (CIs) were calculated for categorical variables. The investigation also included an evaluation of clinical and statistical coherence. Clinical coherence was examined qualitatively by identifying commonalities in interventions, while statistical coherence was tested using loop and design inconsistency tests. Loop consistency utilized node splitting for direct versus indirect treatment effect comparison with significant inconsistency flagged by *P* < .05. Design consistency in network meta-analysis was evaluated, including a global test for inconsistency where *P* < .05 indicated significant deviation from the assumption.

Further qualitative evaluations encompassed the aspects of directness, heterogeneity, and transitivity, ensuring they were in line with the study’s assumptions. The odds ratios (ORs) and 95% confidence intervals (CIs) were obtained using pairwise and network meta-analyses. A random effects model was used, and statistical significance was determined by a two-sided *p*-value of ≤ 0.05. Treatment strategy efficacy was ranked using the surface under the cumulative ranking curve (SUCRA), indicating the likelihood of each intervention being the most effective.

To evaluate the impact of including studies on rib harvest for rhinoplasty, which typically involves smaller harvest size and lesser extent of dissection, we conducted sensitivity analyses using a stepwise exclusion method. Sensitivity analysis was performed by excluding studies related to rhinoplasty to assess the robustness of the overall findings. This approach allowed us to determine whether the inclusion of rhinoplasty studies significantly influenced the efficacy rankings of the pain management strategies.

## Results

### Characteristics of Included Studies

**Fig. 1:**
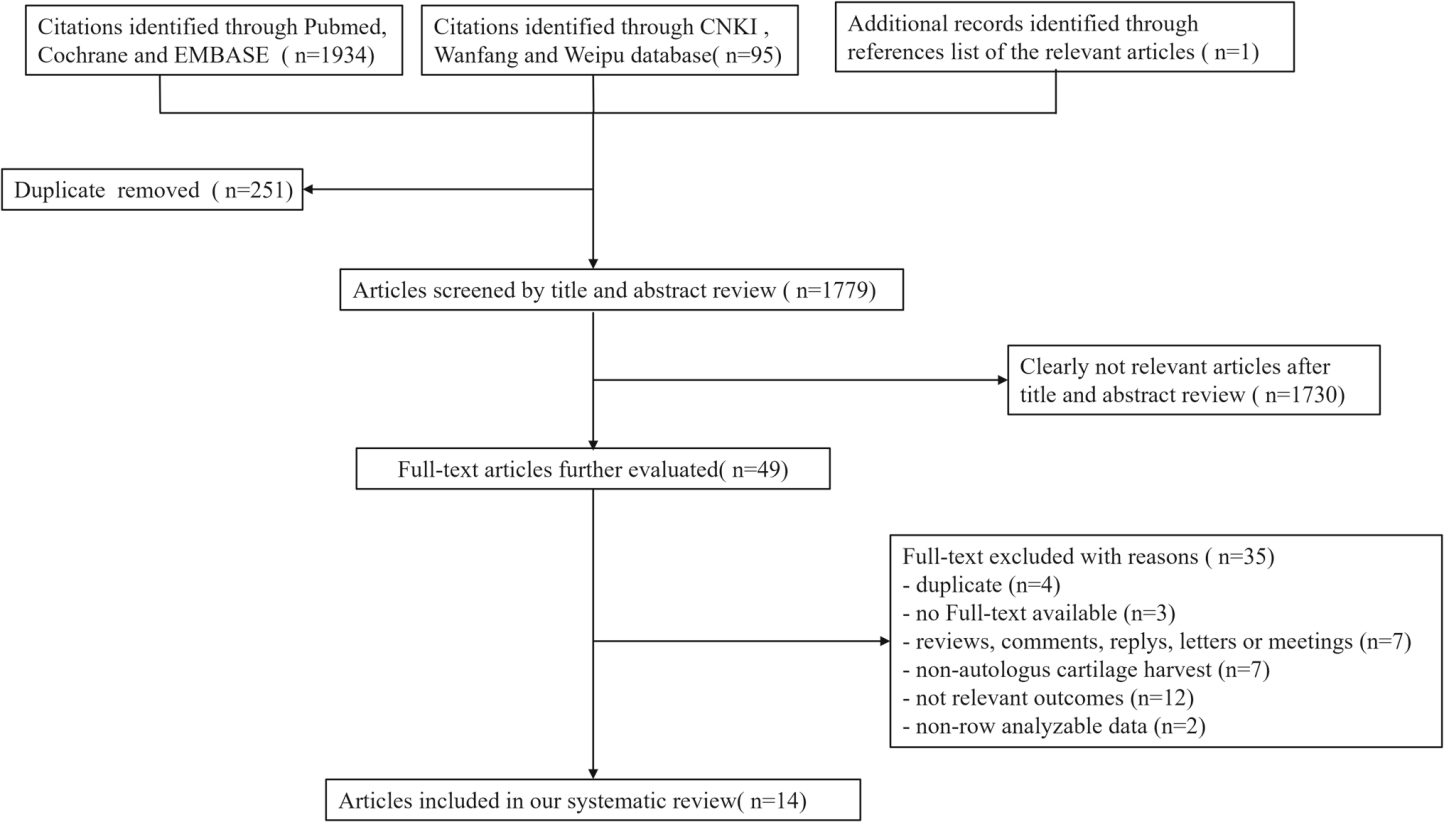
Search flow diagram process for identifying studies eligible for the network meta-analysis

Overall, 2030 records were identified through the initial electronic searches. After removing duplicates, 1779 records were screened for titles and abstracts and 49 full-text articles were screened for eligibility. In total, 14 studies involving 935 participants were included in the review (Fig. 1). Detailed characteristics of the included studies, including study design, participant demographics, interventions, and outcomes, are systematically presented in Table 1.

**Table 1:** Detailed characteristics of the included studies

Publication informations			Patient demographics			Details of the interventions				Clinical outcomes		
Year	Study	Study design	N	Mean age (range)	Sex (M/F)	Timing	Type of intervention	Control groups		Primary outcomes	Period in assessing primary outcome (hours)	Secondary outcomes
2016	Niiyama et al. [14]	RCT	48	11.4	26/22	Post-operation	Ropivacaine CWI	ICNB	N/A	the Face Scale	2,4,12,24,36,48,60,72	Rescue analgesia and adverse effects
2016	Shaffer et al. [15]	Retrospective chart review	15	(4.9-16.1)	7/8	Post-operation	PVB	General anesthesia	N/A	FLACC scale scores	Peak pain score	Rescue analgesia and adverse effects
2017	Chen et al. [20]	RCT	90	(5-10)	65/25	Intra-operative	ICNB +PCA	ICNB	PCA	NRS scores	1,4,6,8,12,24,48	Rescue analgesia and adverse effects
2018	Zhang et al. [8]	RCT	75	(6-14)	48/27	Intra-operative	MB+Ropivacaine ICNB+PCA	ICNB +PCA	PCA	NRS scores	6,24,48,96	Rescue analgesia and adverse effects
2020	Sun et al. [16]	RCT	60	8.84	37/23	Pre-operation	PVB + PCA	PCA	N/A	VAS scores	5min,1,2,4,6,12	Adverse effects
2021	Dong et al. [11]	Prospective study	63	27.78	4/59	Intra-operative	Betamethasone Cocktail ICNB	ropivacaine ICNB	ICNB +PCA	VAS scores	6,12,24,36,48	Rescue analgesia and adverse effects
2022	Chen et al. [17]	RCT	58	(6–9)	41/17	Post-operation	SAPB + PCA	ropivacaine ICNB +PCA	N/A	NRS scores	1,6,12,24,48	Rescue analgesia and adverse effects
2022	Dong et al. [12]	RCT	100	27.87	7/93	Intra-operative	multimodal cocktail intercostal injection	ropivacaine ICNB +PCA	N/A	VAS scores	6,12,24,36,48	Adverse effects
2022	Jiang et al. [9]	RCT	100	(18-40)	0/100	Post-operation	MB & Ropivacaine ICNB + PCA	ICNB + PCA	N/A	VAS scores	6,24,48,72	Rescue analgesia and adverse effects
2022	Liu et al. [10]	RCT	90	10.87	41/49	Intra-operative	MB+Ropivacaine ICNB	MB ICNB	Ropivacaine ICNB	NRS scores	6,24,48,72	Adverse effects
2023	Chen et al. [18]	RCT	60	27.83	7/53	Pre-operation	SAPB & PSB + PCA	SAPB + PCA	N/A	NRS scores	2,4,8,12,24,48	Rescue analgesia and adverse effects
2023	Wang et al. [13]	RCT	66	29.28	5/61	Intra-operative	Triamcinolone Acetonide Cocktail ICNB	Betamethasone Cocktail ICNB	N/A	VAS scores	6,12,24,36,48	Rescue analgesia and adverse effects
2023	Xiang et al. [19]	RCT	60	9.24	37/23	-	Pre-operation SAPB + PCA	Post-operation SAPB + PCA	N/A	NRS scores	1,6,12,24,48	Rescue analgesia and adverse effects
2023	Zheng et al. [21]	RCT	50	(6–12)	25/25	Post-operation	ICNB	General anesthesia	N/A	VAS scores & FLACC scale scores	4,8	Rescue analgesia

### Assessment of the Transitivity, Heterogeneity, and Inconsistency

We evaluated the transitivity assumption and variability among the papers included. Transitivity was verified by consistently employing pain evaluation tools (VAS, FLACC, NRS, Facial Expression Scale) in all therapies, enabling valid indirect comparisons without any observed violations. Despite the various analgesic techniques that were studied, there was an important level of consistency and minimal variation in outcome measures. This can be ascribed to the use of standardized pain rating.

### Risk of Bias

**Fig. 2:**
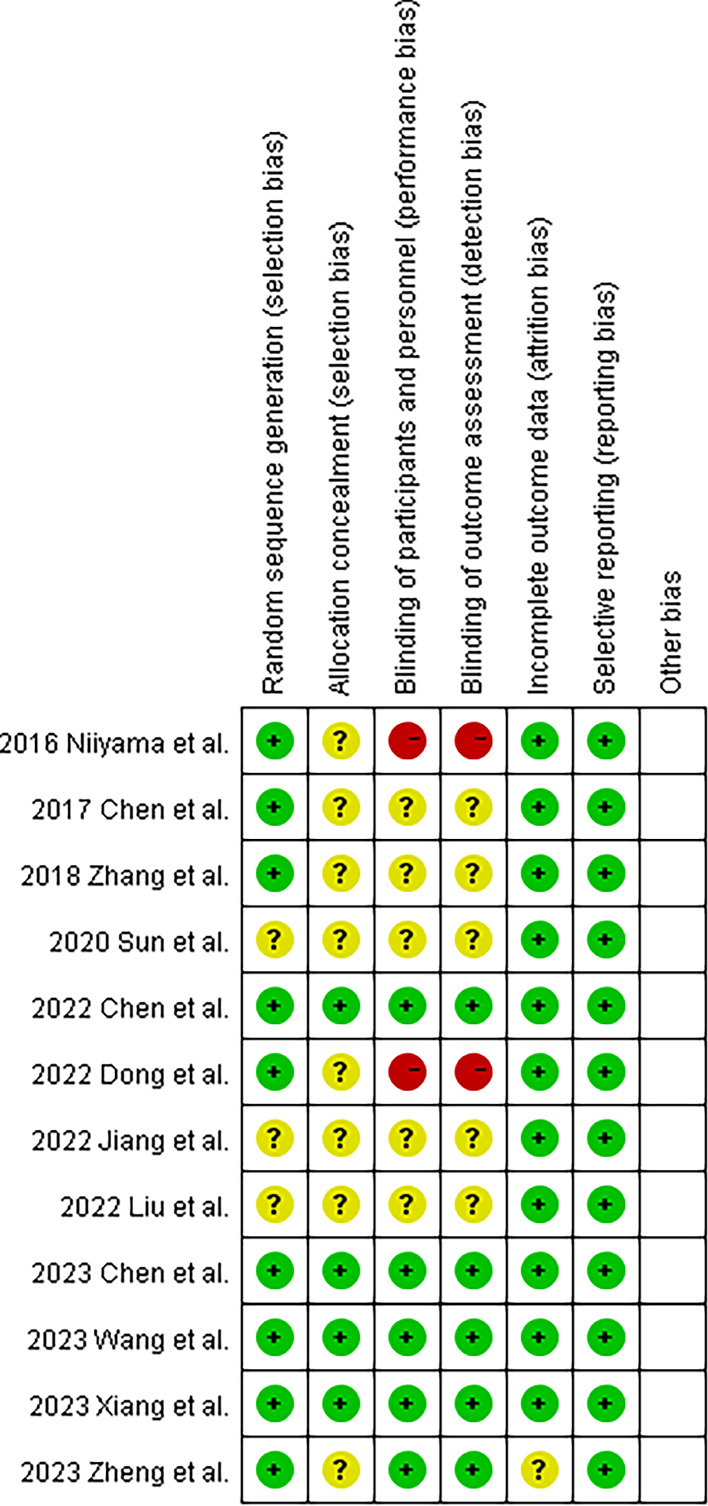
Risk of bias summary. Plus signs indicate minimal risk of bias. Question marks indicate unclear risk. Negative signs indicate considerable risk

The risk of bias for the 12 RCTs was assessed using the RoB-1. Among these studies, 75% adequately addressed randomization, and 91.7% provided complete outcome data, enhancing the credibility of the results. However, only 33.3% demonstrated adequate allocation concealment, and merely 41.7% effectively implemented blinding for participants and outcome assessment, indicating areas for improvement in study design and execution. Notably, all 12 studies exhibited commendable reporting practices with no evidence of selective reporting bias (Fig. 2).

The risk of bias for the two cohort studies was evaluated using the NOS. Both studies scored 4 out of 4 in the selection domain. In the comparability domain, one study scored 1 while the other scored 0. For the exposure/outcome domain, one study scored 2 and the other scored 3. Overall, one study achieved a total score of 6 (indicating moderate risk), while the other scored 8 (indicating low risk) (Table 2).

**Table 2:** Methodological quality score of the included studies based on the Newcastle–Ottawa Scale (NOS) tool

Author	Year	Selection				Comparability	Exposure/outcome			Total Score	Risk of bias
		Representativeness of cohort (*)	Selection of control cohort (*)	Ascertainment of exposure (*)	Outcome not- present at start (*)	Comparability of cohorts (**)	Assessment of outcome (*)	Length of follow-up (*)	Adequacy of follow-up (*)	Total score 9*	
Shaffer et al.	2016	*	*	*	*		*		*	6	Moderate
Dong et al.	2021	*	*	*	*	*	*	*	*	8	Low

### The Results of Network Meta-analysis

#### Primary Outcome

**Fig. 3:**
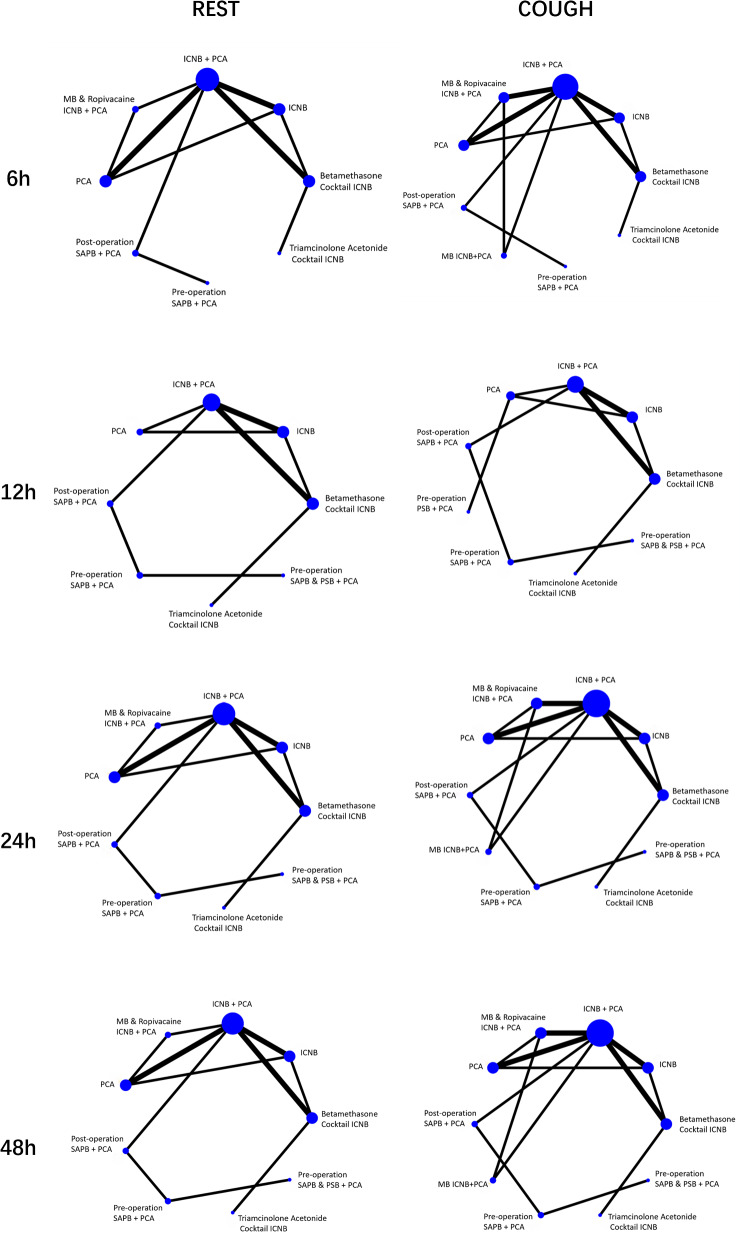
Pain scores network plots. The size of the treatment nodes reflects the proportionate numbers of patients assigned to the treatment group, whereas the edge thickness is proportional to the number of studies supporting each comparison

The 6 h, 12 h, 24 h and 48 h postoperative pain scores during rest and coughing is the primary outcome of this study. Figure 3 illustrates the direct comparisons and the distribution of sample sizes among various local anesthetic interventions.

##### Resting Pain Scores

**Fig. 4:**
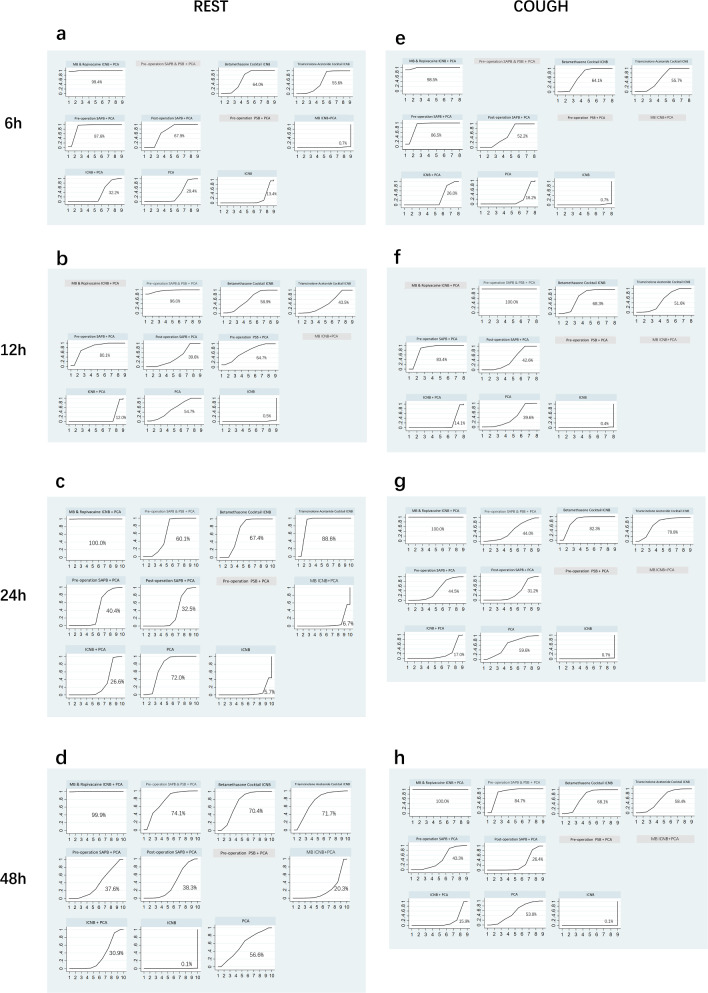
SUCRA efficacy rate ranking curve for pain scores. Analgesic techniques are being compared based on the pain scores at different timepoints during rest or coughing, using the cumulative ranking area (SUCRA). The higher the probability, the lower the pain score, and the more potent the analgesic benefits

Eight studies with 596 participants were examined to assess the pain levels during the resting period six hours after surgery. The network meta-analysis revealed that the most effective local anesthetic interventions for reducing postoperative 6-hour resting pain scores were MB & Ropivacaine ICNB + PCA, Pre-operation SAPB + PCA, and Post-operation SAPB + PCA. These interventions had SUCRA rankings of 99.4%, 87.6%, and 67.9%, respectively. In contrast, the MB ICNB+PCA method was shown to be the least effective, with a ranking of only 0.7% (Fig. 4a, Table 3a).

**Table 3:** Comparisons of analgesic methods using network meta-analysis

a. League tables of resting pain score at 6h postoperative								
MB & Ropivacaine ICNB + PCA								
**− **0.91 (**− **1.99, 0.16)	Pre-operation SAPB + PCA							
**− 1.74 (− 2.67, − 0.81)**	**− 0.83 (− 1.36, − 0.30)**	Post-operation SAPB + PCA						
**− 1.01 (− 1.90, − 0.11)**	**4.18 (2.92, 5.43)**	**2.97 (2.07, 3.87)**	Betamethasone Cocktail ICNB					
**− 2.09 (− 3.16, − 1.02)**	**− 1.18 (− 2.27, − 0.09)**	**− 0.35 (− 1.30, 0.60)**	**− 1.19 (− 2.07, − 0.31)**	Triamcinolone Acetonide Cocktail ICNB				
**− 2.97 (− 3.72, − 2.23)**	**− 2.06 (− 2.84, − 1.29)**	**− 1.23 (− 1.80, − 0.67)**	**− 5.77 (− 6.99, − 4.54)**	**− 0.88 (− 1.65, − 0.12)**	ICNB + PCA			
**− 3.11 (− 4.25, − 1.97)**	**− 2.20 (− 3.36, − 1.04)**	**− 1.37 (− 2.41, − 0.34)**	**− 1.05 (− 1.64, − 0.46)**	**− 1.02 (− 2.03, − 0.02)**	**− **0.14 (**− **1.00, 0.73)	PCA		
**− 3.53 (− 4.55, − 2.51)**	**− 2.62 (− 3.67, − 1.58)**	**− 1.79 (− 2.69, − 0.90)**	**− 1.61 (− 2.33, − 0.90)**	**− 1.44 (− 2.31, − 0.58)**	**− **0.56 (**− **1.26, 0.14)	**− **0.42 (**− **0.93, 0.09)	ICNB	
**− 4.31 (− 5.23, − 3.39)**	**− 3.39 (− 4.34, − 2.45)**	**− 2.57 (− 3.35, − 1.78)**	**1.92 (0.97, 2.87)**	**− 2.22 (− 3.16, − 1.28)**	**− 1.33 (− 1.88, − 0.79)**	**− 1.19 (− 2.22, − 0.17)**	**− **0.77 (**− **1.66, 0.11)	MB ICNB+PCA

b. League tables of resting pain score at 12h postoperative								
Pre-operation SAPB & PSB + PCA								
**− **0.42 (**− **0.93, 0.09)	Pre-operation SAPB + PCA							
**− **0.86 (**− **2.27, 0.54)	**− **0.44 (**− **1.75, 0.87)	Pre-operation PSB + PCA						
**− **0.58 (**− **1.58, 0.42)	0.32 (**− **0.53, 1.17)	0.01 (**− **0.97, 0.99)	Betamethasone Cocktail ICNB					
**− **1.01 (**− **2.32, 0.30)	**− **0.59 (**− **1.80, 0.62)	**− **0.15 (**− **0.65, 0.36)	**3.08 (2.06, 4.11)**	PCA				
**− **1.23 (**− **2.45, **− **0.00)	**− **0.81 (**− **1.92, 0.31)	**− **0.36 (**− **1.57, 0.84)	0.14 (**− **0.96, 1.24)	**− **0.22 (**− **1.31, 0.87)	Triamcinolone Acetonide Cocktail ICNB			
**− 1.32 (− 2.06, − 0.58)**	**− 0.90 (− 1.43, − 0.37)**	**− **0.46 (**− **1.65, 0.74)	**− 1.59 (− 2.22, − 0.96)**	**− **0.31 (**− **1.40, 0.78)	**− **0.09 (**− **1.07, 0.89)	Post-operation SAPB + PCA		
**− 2.59 (− 3.52, − 1.66)**	**− 2.17 (− 2.95, − 1.39)**	**− 1.73 (− 2.78, − 0.67)**	**− 4.80 (− 6.00, − 3.61)**	**− 1.58 (− 2.51, − 0.65)**	**− 1.36 (− 2.16, − 0.57)**	**− 1.27 (− 1.84, − 0.70)**	ICNB + PCA	
**− 3.20 (− 4.37, − 2.04)**	**− 2.78 (− 3.83, − 1.73)**	**− 2.34 (− 3.13, − 1.55)**	**− 2.20 (− 2.97, − 1.44)**	**− 2.19 (− 2.80, − 1.59)**	**− 1.97 (− 2.88, − 1.07)**	**− 1.88 (− 2.78, − 0.98)**	**− **0.61 (**− **1.31, 0.09)	ICNB

c. League tables of resting pain score at 24h postoperative									
MB & Ropivacaine ICNB + PCA									
**− 1.57 (− 2.79, − 0.35)**	TriamcinoloneAcetonide Cocktail ICNB								
**− 2.68 (− 3.98, − 1.38)**	**− **1.11 (**− **2.22, 0.00)	PCA							
**− 3.56 (− 4.64, − 2.49)**	**− **0.20 (**− **1.18, 0.77)	1.42 (0.80, 2.04)	Betamethasone Cocktail ICNB						
**− 3.25 (− 4.52, − 1.98)**	**− 1.68 (− 2.89, − 0.47)**	**− **0.57 (**− **1.86, 0.72)	**− 1.25 (− 2.05, − 0.44)**	Pre-operation SAPB & PSB + PCA					
**− 3.92 (− 5.08, − 2.76)**	**− 2.35 (− 3.44, − 1.26)**	**− 1.24 (− 2.42, − 0.06)**	**− 2.24 (− 3.33, − 1.15)**	**− 0.67 (− 1.19, − 0.15)**	Pre-operation SAPB + PCA				
**− 4.13 (− 5.17, − 3.09)**	**− 2.56 (− 3.52, − 1.59)**	**− 1.45 (− 2.52, − 0.38)**	**− 3.79 (− 4.83, − 2.75)**	**− 0.88 (− 1.60, − 0.15)**	**− **0.21 (**− **0.72, 0.30)	Post-operation SAPB + PCA			
**− 4.30 (− 5.21, − 3.40)**	**− 2.73 (− 3.55, − 1.92)**	**− 1.62 (− 2.56, − 0.69)**	**− 5.41 (− 6.64, − 4.17)**	**− 1.05 (− 1.94, − 0.16)**	**− **0.39 (**− **1.11, 0.34)	**− **0.17 (**− **0.69, 0.34)	ICNB + PCA		
**− 4.91 (− 5.95, − 3.86)**	**− 3.34 (− 4.30, − 2.37)**	**− 2.23 (− 3.29, − 1.16)**	**2.88 (1.79, 3.98)**	**− 1.66 (− 2.68, − 0.63)**	**− 0.99 (− 1.88, − 0.10)**	**− 0.78 (− 1.51, − 0.05)**	**− 0.60 (− 1.12, − 0.09)**	MB ICNB +PCA	
**− 4.98 (− 6.13, − 3.84)**	**− 3.41 (− 4.34, − 2.48)**	**− 2.30 (− 2.92, − 1.69)**	**− 2.10 (− 2.86, − 1.34)**	**− 1.73 (− 2.87, − 0.60)**	**− 1.06 (− 2.07, − 0.06)**	-0.85 (-1.72,0.02)	**− **0.68 (**− **1.38, 0.02)	**− **0.08 (**− **0.95, 0.80)	ICNB

d. League tables of resting pain score at 48h postoperative									
MB & Ropivacaine ICNB + PCA									
**− 1.41 (− 2.52, − 0.30)**	Pre-operation SAPB & PSB + PCA								
**− 1.48 (− 2.49, − 0.48)**	**− **0.07 (**− **1.23, 1.10)	Triamcinolone Acetonide Cocktail ICNB							
**− 2.63 (− 3.71, − 1.56)**	0.56 (**− **0.21, 1.33)	0.23 (**− **0.71, 1.18)	Betamethasone Cocktail ICNB						
**− 1.75 (− 2.90, − 0.61)**	-0.34 (-1.63,0.95)	**− **0.27 (**− **1.33, 0.79)	**− 0.67 (− 1.25, − 0.10)**	PCA					
**− 2.08 (− 2.92, − 1.24)**	**− **0.67 (**− **1.39, 0.06)	**− **0.60 (**− **1.51, 0.31)	**− 1.07 (− 1.92, − 0.22)**	**− **0.33 (**− **1.39, 0.74)	Post-operation SAPB + PCA				
**− 2.08 (− 3.06, − 1.10)**	**− 0.67 (− 1.19, − 0.15)**	**− **0.60 (**− **1.64, 0.44)	**− **0.71 (**− **1.80, 0.37)	**− **0.33 (**− **1.51, 0.85)	0.00 (**− **0.51, 0.51)	Pre-operation SAPB + PCA			
**− 2.20 (− 2.86, − 1.53)**	**− **0.78 (**− **1.67, 0.11)	**− **0.71 (**− **1.46, 0.04)	**-4.40 (− 5.42, − 3.38)**	**− **0.44 (**− **1.38, 0.49)	**− **0.12 (**− **0.63, 0.40)	**− **0.12 (**− **0.84, 0.61)	ICNB + PCA		
**− 2.40 (− 3.24, − 1.57)**	**− **0.99 (**− **2.02, 0.03)	**− 0.92 (− 1.83, − 0.02)**	**1.52 (0.64, 2.40)**	**− **0.65 (**− **1.71, 0.41)	**− **0.32 (**− **1.05, 0.40)	**− **0.32 (**− **1.21, 0.56)	**− **0.21 (**− **0.72, 0.30)	MB ICNB +PCA	
**− 3.56 (− 4.55, − 2.57)**	**− 2.15 (− 3.30, − 0.99)**	**− 2.08 (− 2.97, − 1.19)**	**− 2.04 (− 2.79, − 1.29)**	**− 1.81 (− 2.38, − 1.23)**	**− 1.48 (− 2.38, − 0.58)**	**− 1.48 (− 2.51, − 0.45)**	**− 1.37 (− 2.10, − 0.63)**	**− 1.16 (− 2.05, − 0.26)**	ICNB

e. League tables of coughing pain score at 6h postoperative							
MB & Ropivacaine ICNB + PCA							
**− **0.72 (**− **1.84, 0.40)	Pre-operation SAPB + PCA						
**− 1.53 (− 2.48, − 0.58)**	**2.76 (1.60, 3.93)**	Betamethasone Cocktail ICNB					
**− 1.91 (− 3.02, − 0.79)**	**− 1.18 (− 2.31, − 0.06)**	**− 1.97 (− 2.92, − 1.03)**	Triamcinolone Acetonide Cocktail ICNB				
**− 2.04 (− 3.01, − 1.07)**	**− 1.32 (− 1.88, − 0.75)**	**1.76 (0.76, 2.76)**	**− **0.13 (**− **1.11, 0.84)	Post-operation SAPB + PCA			
**− 3.31 (− 4.10, − 2.53)**	**− 2.59 (− 3.39, − 1.79)**	**− 7.06 (− 8.52, − 5.60)**	**− 1.40 (− 2.20, − 0.61)**	**− 1.27 (− 1.84, − 0.70)**	ICNB + PCA		
**− 3.73 (− 4.91, − 2.56)**	**− 3.01 (− 4.20, − 1.83)**	**− 1.55 (− 2.18, − 0.92)**	**− 1.83 (− 2.88, − 0.77)**	**− 1.70 (− 2.74, − 0.65)**	**− **0.42 (**− **1.30, 0.45)	PCA	
**− 4.20 (− 5.26, − 3.14)**	**− 3.48 (− 4.55, − 2.41)**	**− 2.44 (− 3.23, − 1.65)**	**− 2.29 (− 3.22, − 1.37)**	**− 2.16 (− 3.07, − 1.25)**	**− 0.89 (− 1.60, − 0.18)**	**− **0.47 (**− **0.98, 0.05)	ICNB

f. League tables of coughing pain score at 12h postoperative							
Pre-operation SAPB & PSB + PCA							
**− 1.33 (− 1.90, − 0.77)**	Pre-operation SAPB + PCA						
**− **0.68 (**− **1.69, 0.33)	0.67 (**− **0.30, 1.63)	Betamethasone Cocktail ICNB					
**− 2.30 (− 3.56, − 1.05)**	**− **0.97 (**− **2.09, 0.15)	**− **0.55 (**− **1.39, 0.30)	Triamcinolone Acetonide Cocktail ICNB				
**− 2.56 (− 3.35, − 1.77)**	**− 1.23 (− 1.78, − 0.67)**	**− 1.65 (− 2.29, − 1.02)**	**− **0.25 (**− **1.23, 0.72)	Post-operation SAPB + PCA			
**− 2.68 (− 4.00, − 1.36)**	**− 1.35 (− 2.54, − 0.15)**	**2.69 (1.68, 3.69)**	**− **0.37 (**− **1.45, 0.71)	**− **0.12 (**− **1.18, 0.94)	PCA		
**− 3.67 (− 4.63, − 2.70)**	**− 2.33 (− 3.12, − 1.55)**	**− 6.87 (− 8.33, − 5.42)**	**− 1.36 (− 2.16, − 0.56)**	**− 1.11 (− 1.67, − 0.55)**	**− 0.99 (− 1.89, − 0.09)**	ICNB + PCA	
**− 4.34 (− 5.54, − 3.15)**	**− 3.01 (− 4.06, − 1.96)**	**− 2.33 (− 3.11, − 1.55)**	**− 2.04 (− 2.96, − 1.12)**	**− 1.78 (− 2.68, − 0.89)**	**− 1.66 (− 2.23, − 1.10)**	**− **0.68 (**− **1.38, 0.03)	ICNB

g. League tables of coughing pain score at 24h postoperative								
MB & Ropivacaine ICNB + PCA								
**− 2.34 (− 3.32, − 1.35)**	Betamethasone Cocktail ICNB							
**− 3.06 (− 4.26, − 1.86)**	**− **0.52 (**− **1.47, 0.43)	Triamcinolone Acetonide Cocktail ICNB						
**− 3.38 (− 4.65, − 2.10)**	**− 1.43 (− 2.05, − 0.81)**	-0.32 (-1.38,0.75)	PCA					
**− 3.79 (− 4.95, − 2.63)**	**− 1.27 (− 2.34, − 0.21)**	**− **0.73 (**− **1.80, 0.34)	**− **0.41 (**− **1.57, 0.75)	Pre-operation SAPB + PCA				
**− 3.79 (− 5.05, − 2.52)**	**− 1.14 (− 1.95, − 0.34)**	**− **0.73 (**− **1.91, 0.45)	**− **0.41 (**− **1.67, 0.85)	0.00 (**− **0.51, 0.51)	Pre-operation SAPB & PSB + PCA			
**− 4.00 (− 5.04, − 2.96)**	**2.85 (1.76, 3.95)**	**− **0.94 (**− **1.88, 0.00)	**− 0.62 (− 1.66, 0.42)**	**− **0.21 (**− **0.72, 0.30)	**− **0.21 (**− **0.93, 0.51)	Post-operation SAPB + PCA		
**− 4.28 (− 5.19, − 3.38)**	**− 8.15 (− 9.73, − 6.57)**	**− 1.23 (− 2.01, − 0.44)**	**− 0.91 (− 1.81, − 0.01)**	**− **0.50 (**− **1.22, 0.23)	**− **0.50 (**− **1.38, 0.39)	**− **0.29 (**− **0.81, 0.23)	ICNB + PCA	
**− 5.03 (− 6.18, − 3.88)**	**− 2.18 (− 2.94, − 1.41)**	**− 1.97 (− 2.88, − 1.07)**	**− 1.65 (− 2.22, − 1.09)**	**− 1.24 (− 2.25, − 0.23)**	**− 1.24 (− 2.37, − 0.11)**	**− 1.03 (− 1.91, − 0.16)**	**− 0.75 (− 1.45, − 0.04)**	ICNB

h. League tables of coughing pain score at 48h postoperative								
MB & Ropivacaine ICNB + PCA								
**− 2.22 (− 3.49, − 0.95)**	Pre-operation SAPB & PSB + PCA							
**− 2.58 (− 3.61, − 1.56)**	**0.92 (0.13, 1.71)**	Betamethasone Cocktail ICNB						
**− 3.10 (− 4.28, − 1.93)**	**− **0.89 (**− **2.08, 0.31)	**− **0.31 (**− **1.26, 0.65)	Triamcinolone Acetonide Cocktail ICNB					
**− 3.26 (− 4.53, − 1.99)**	**− **1.04 (**− **2.34, 0.26)	**− **1.16 (**− **1.76, **− **0.56)	**− **0.16 (**− **1.22, 0.91)	**PCA**				
**− 3.55 (− 4.69, − 2.41)**	**− 1.33 (− 1.90, − 0.77)**	**− **1.01 (**− **2.08, 0.06)	**− **0.45 (**− **1.51, 0.61)	**− **0.29 (**− **1.46, 0.88)	Pre-operation SAPB + PCA			
**− 3.88 (− 4.90, − 2.86)**	**− 1.66 (− 2.42, − 0.90)**	**2.96 (1.89,4.02)**	**− **0.77 (**− **1.70, 0.15)	**− **0.62 (**− **1.67, 0.44)	**− **0.33 (**− **0.84, 0.18)	Post-operation SAPB + PCA		
**− 4.12 (− 5.00, − 3.24)**	**− 1.90 (− 2.82, − 0.98)**	**− 6.00 (− 7.27, − 4.73)**	**− 1.01 (− 1.78, − 0.24)**	**− **0.86 (**− **1.77, 0.06)	**− **0.57 (**− **1.29, 0.16)	**− **0.24 (**− **0.76, 0.28)	ICNB + PCA	
**-5.08 (− 6.22, − 3.95)**	**− 2.87 (− 4.03, − 1.70)**	**− 2.13 (− 2.89, − 1.37)**	**− 1.98 (− 2.88, − 1.08)**	**− 1.82 (− 2.40, − 1.25)**	**− 1.53 (− 2.55, − 0.52)**	**− 1.21 (− 2.09, − 0.33)**	**− 0.97 (− 1.68, − 0.25)**	ICNB

*The data represents the relative risks (with a 95% confidence interval) of the column-defining therapy compared to the row-defining treatment. Column-defining technique is used when the RRs are greater than 0. RRs below 0 indicate a preference for the row-defining procedure. Note worthy findings are displayed in bold type.

For postoperative 12-hour resting pain scores, eight studies with 551 participants were analyzed. The analysis showed that Pre-operation SAPB & PSB + PCA, Pre-operation SAPB + PCA, and Pre-operation PSB + PCA were the most efficacious interventions, with SUCRA rankings of 96.0%, 80.1%, and 64.7%, respectively. Traditional single drug ICNB was ranked as the least effective, with a SUCRA score of 0.5% (Fig. 4b, Table 3b).

The analysis focused on the 24-h postoperative resting pain scores of 656 participants from nine studies. The three most effective therapies identified were MB & Ropivacaine ICNB + PCA, Triamcinolone Acetonide Cocktail ICNB, and PCA, with SUCRA scores of 100%, 88.6%, and 72.0%, respectively. The intervention that had the lowest effectiveness was the typical single medication ICNB, as shown by an SUCRA score of 5.7% (Fig. 4c, Table 3c).

Regarding the 48-h postoperative resting pain scores, our analysis of nine studies with 656 participants found that the most effective interventions were MB & Ropivacaine ICNB + PCA, Pre-operation SAPB & PSB + PCA, and Triamcinolone Acetonide Cocktail ICNB, with SUCRA scores of 99.9%, 74.1%, and 71.7%, respectively. Traditional single drug ICNB was the least effective, with a SUCRA score of 0.1% (Fig. 4d, Table 3d).

##### Cough Pain Scores

The analysis focused on the cough pain levels of 506 individuals from seven studies, specifically looking at the values recorded six hours after the surgery. The results emphasized the effectiveness range among the assessed local anesthetic categories. The primary therapies were MB & Ropivacaine ICNB + PCA, Pre-operation SAPB + PCA, and Betamethasone Cocktail ICNB, with SUCRA scores of 98.5%, 86.5%, and 64.1%, respectively. The intervention traditional ICNB with single drug was found to be the least effective, with a SUCRA score of 0.7% (Fig. 4e, Table 3e).

For postoperative 12-hour cough pain scores, seven studies with 491 participants were analyzed. According to the network meta-analysis, the interventions leading in effectiveness were Pre-operation SAPB & PSB + PCA, Pre-operation SAPB + PCA, and Betamethasone Cocktail ICNB, with SUCRA scores of 100%, 83.4%, and 68.3%, respectively. Traditional single drug ICNB was the least effective, with a SUCRA score of 0.4% (Fig. 4f, Table 3f).

Eight studies, comprising 536 participants, were examined to determine the 24-hour postoperative cough pain levels. The network meta-analysis revealed that the most effective therapies were MB & Ropivacaine ICNB + PCA, Betamethasone Cocktail ICNB, and Triamcinolone Acetonide Cocktail ICNB, with corresponding SUCRA ratings of 100%, 82.3%, and 70.8%. The least efficient approach was the use of standard ICNB with a single medication, which had an SUCRA ranking of only 0.7%. (Fig. 4g, Table 3g).

For the 48-hour postoperative cough pain scores, the analysis of eight studies with 536 participants identified MB & Ropivacaine ICNB + PCA, Pre-operation SAPB & PSB + PCA, and Betamethasone Cocktail ICNB as the top interventions, with SUCRA scores of 100%, 84.7%, and 68.1%, respectively. Traditional ICNB with single drug remained the least effective, with a SUCRA score of 0.1% (Fig. 4h, Table 3h).

#### Secondary Outcomes

**Fig. 5:**
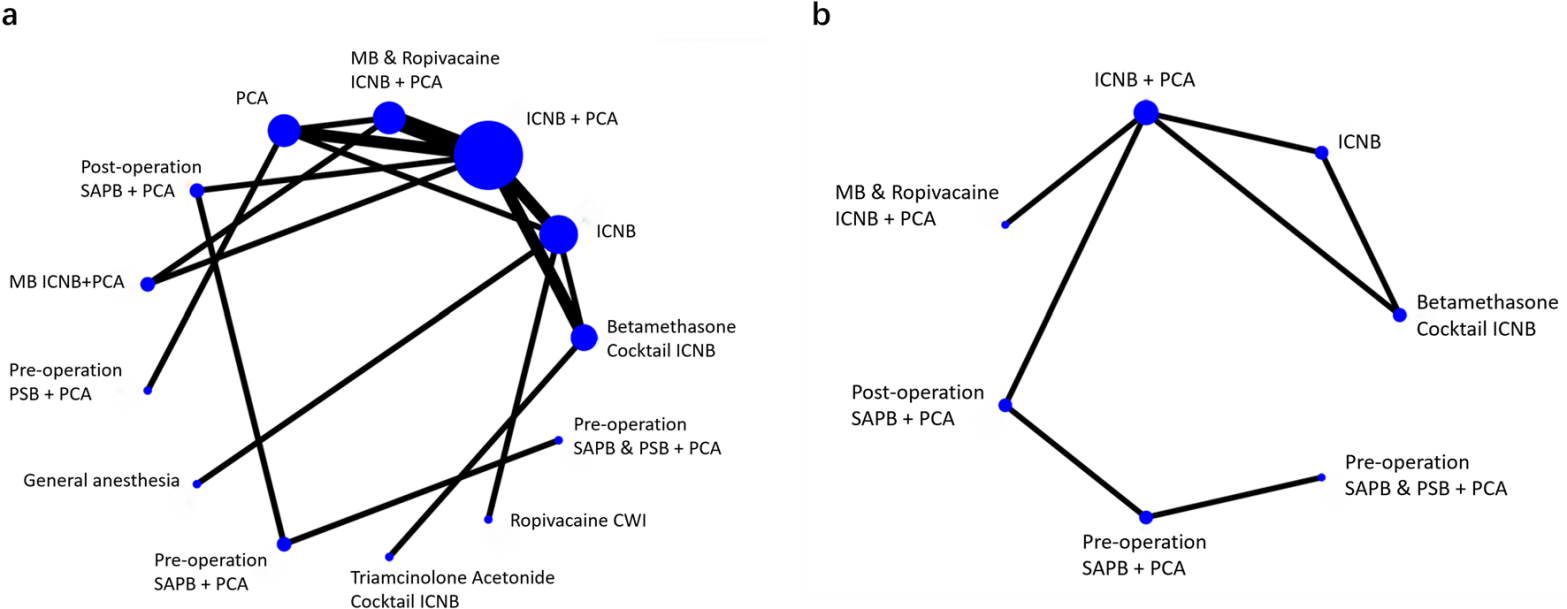
Secondary outcomes network plots. **a**. The incidence of opioid-related adverse effects network plots. **b**. The utilization of rescue analgesia network plots

**Fig. 6:**
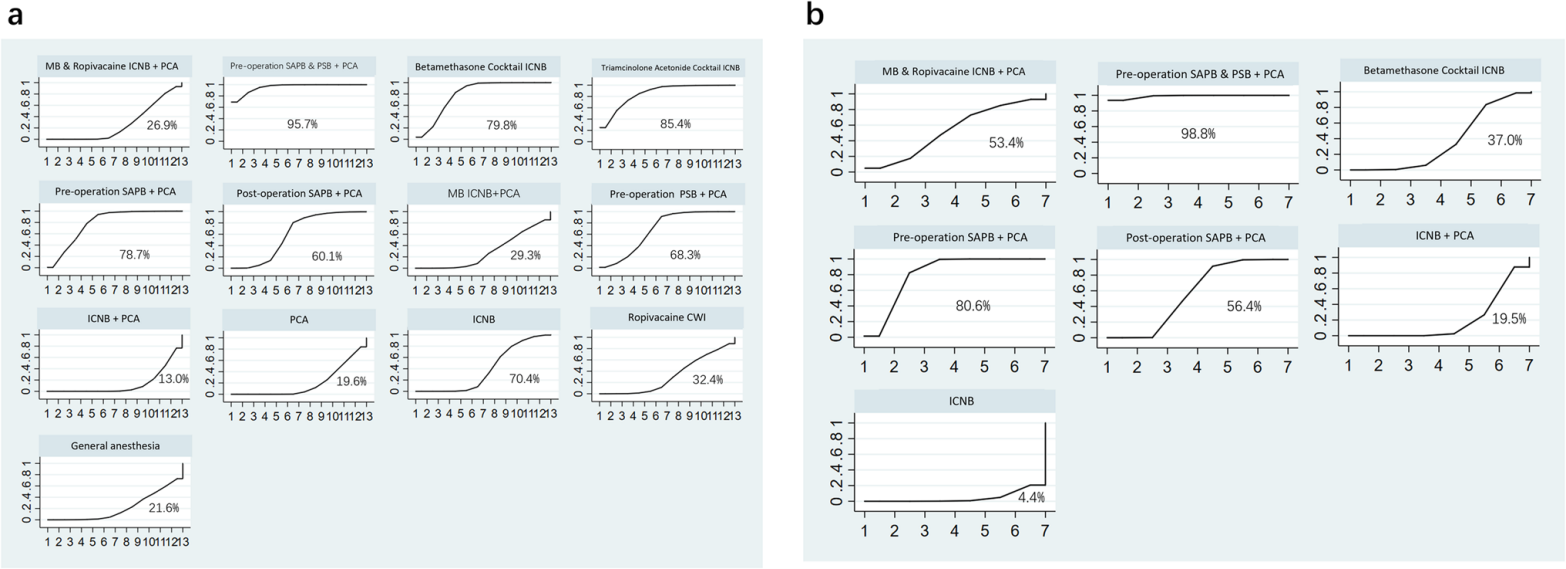
SUCRA efficacy rate ranking curve for secondary outcomes. **a**. The incidence of opioid-related adverse effects SUCRA ranking, larger the probability, lower the incidence of adverse effects. **b**. The utilization of rescue analgesia SUCRA ranking, larger the probability, less the utilization of rescue analgesia, stronger the analgesic effects

Thirteen studies including 914 people were used to investigate the occurrence of problems connected to opioids. Figure 5a presents an analysis that visually shows how diverse types of local anesthetics work in reducing the negative effects caused by opioids. The three most effective therapies were Pre-operation SAPB & PSB + PCA, Triamcinolone Acetonide Cocktail ICNB, and Betamethasone Cocktail ICNB, with SUCRA ratings of 95.7%, 85.4%, and 79.8%, respectively. The intervention that had the lowest success in minimizing the occurrence of problems associated with opioids was the typical single medication ICNB + PCA, as shown by an SUCRA score of 13.0% (Fig. 6a, Table 4a).

**Table 4:** League tables of secondary outcomes

a. League tables of the incidence of opioid-related adverse effects												
Pre-operation SAPB & PSB + PCA												
0.36 (0.01, 10.51)	Triamcinolone Acetonide Cocktail ICNB											
0.22 (0.01, 3.64)	0.60 (0.09, 3.87)	Betamethasone Cocktail ICNB										
0.20 (0.04, 1.02)	0.55 (0.03, 10.60)	0.91 (0.09, 9.09)	Pre-operation SAPB + PCA									
0.10 (0.01, 1.74)	0.27 (0.02, 4.18)	0.45 (0.06, 3.33)	0.50 (0.05, 5.29)	Pre-operation PSB + PCA								
**0.07 (0.01, 0.52)**	0.19 (0.01, 2.82)	0.32 (0.04, 2.22)	0.35 (0.10, 1.16)	0.69 (0.09, 5.25)	Post-operation SAPB + PCA							
**0.02 (0.00, 0.34)**	**0.06 (0.01, 0.80)**	**0.11 (0.02, 0.59)**	**0.12 (0.01, 0.99)**	0.23 (0.05, 1.18)	0.33 (0.06, 1.96)	ICNB						
**0.02 (0.00, 0.36)**	**0.05 (0.00, 0.86)**	**0.09 (0.01, 0.70)**	0.10 (0.01, 1.11)	0.20 (0.03, 1.43)	0.28 (0.03, 2.32)	0.84 (0.27, 2.64)	Ropivacaine CWI					
**0.02 (0.00, 0.29)**	**0.05 (0.00, 0.69)**	**0.08 (0.01, 0.53)**	**0.09 (0.01, 0.86)**	0.18 (0.03, 1.21)	0.25 (0.04, 1.74)	0.76 (0.14, 4.07)	0.90 (0.12, 6.85)	MB ICNB +PCA				
**0.02 (0.00, 0.22)**	**0.04 (0.00, 0.51)**	**0.07 (0.01, 0.36)**	**0.08 (0.01, 0.62)**	**0.16 (0.03, 0.79)**	0.23 (0.04, 1.20)	0.69 (0.19, 2.59)	0.82 (0.14, 4.69)	0.92 (0.24, 3.44)	MB & Ropivacaine ICNB + PCA			
**0.01 (0.00, 0.26)**	**0.04 (0.00, 0.62)**	**0.06 (0.01, 0.50)**	**0.07 (0.01, 0.79)**	0.14 (0.02, 1.02)	0.20 (0.02, 1.65)	0.60 (0.19, 1.90)	0.71 (0.14, 3.59)	0.79 (0.10, 6.08)	0.86 (0.15, 4.96)	General anesthesia		
**0.01 (0.00, 0.18)**	**0.04 (0.00, 0.42)**	**0.06 (0.01, 0.30)**	**0.07 (0.01, 0.51)**	**0.13 (0.04, 0.45)**	**0.19 (0.04, 0.98)**	0.58 (0.19, 1.72)	0.68 (0.14, 3.32)	0.76 (0.17, 3.43)	0.83 (0.29, 2.35)	0.96 (0.20, 4.72)	PCA	
**0.01 (0.00, 0.13)**	**0.03 (0.00, 0.31)**	**0.05 (0.01, 0.20)**	**0.06 (0.01, 0.36)**	**0.11 (0.03, 0.49)**	**0.16 (0.04, 0.67)**	0.49 (0.17, 1.43)	0.58 (0.12, 2.77)	0.65 (0.17, 2.42)	0.70 (0.30, 1.65)	0.82 (0.17, 3.94)	0.85 (0.37, 1.93)	ICNB + PCA

b. League tables of the utilization of rescue analgesia						
Pre-operation SAPB & PSB + PCA						
**0.22 (0.05, 0.91)**	Pre-operation SAPB + PCA					
**0.05 (0.01, 0.27)**	**0.20 (0.07, 0.62)**	Post-operation SAPB + PCA				
0.04 (0.00, 1.76)	**0.18 (0.01, 6.02)**	0.89 (0.03, 24.68)	MB & Ropivacaine ICNB + PCA			
**0.01 (0.00, 0.20)**	**0.07 (0.01, 0.59)**	0.33 (0.05, 2.12)	0.37 (0.01, 10.48)	Betamethasone Cocktail ICNB		
**0.01 (0.00, 0.07)**	**0.03 (0.01, 0.19)**	**0.17 (0.05, 0.62)**	0.19 (0.01, 4.10)	0.52 (0.13, 2.01)	ICNB + PCA	
**0.00 (0.00, 0.05)**	**0.02 (0.00, 0.15)**	**0.08 (0.01, 0.55)**	0.09 (0.00, 2.65)	0.24 (0.06, 1.04)	0.47 (0.11, 1.98)	ICNB

*Data are RRs (95% CI) in the column-defining treatment compared with the row-defining treatment. RRs higher than 1 favor the column-defining treatment. RRs lower than 1 favor the row-defining treatment. Significant results are in bold.

For the utilization of rescue analgesia, five studies comprising 444 participants were analyzed. The analysis, displayed in Fig. 5b, provided a direct comparison and distribution of sample sizes across different local anesthetic interventions. The network meta-analysis revealed that the most effective interventions in reducing the incidence of rescue analgesia use were Pre-operation SAPB & PSB + PCA, Pre-operation SAPB + PCA, and Post-operation SAPB + PCA, with their SUCRA scores, respectively, at 98.8%, 80.6%, and 56.4%. The least effective intervention was identified as traditional ICNB with single drug, with a SUCRA ranking of 4.4% (Fig. 6b, Table 4b).

### Sensitivity Analysis

To evaluate the impact of including studies on rib harvest for rhinoplasty, which typically involves a smaller harvest size and lesser extent of dissection, we conducted a sensitivity analysis using a stepwise exclusion method. The primary and secondary outcomes were re-evaluated to assess the robustness of the overall findings. The exclusion of rhinoplasty studies did not significantly alter the rankings or conclusions of the pain management strategies. Multimodal analgesic strategies, particularly “Methylene Blue and Ropivacaine Intercostal Nerve Block (MB & Ropivacaine ICNB) combined with Patient-Controlled Analgesia (PCA)”, and “Pre-operation SAPB & PSB + PCA”, remained the most effective approaches for reducing postoperative pain and minimizing the need for rescue analgesia and opioid-related adverse effects.

These results confirm that the inclusion of studies on rib harvest for rhinoplasty does not significantly influence the overall conclusions of our systematic review and network meta-analysis. Multimodal analgesic strategies provide superior pain control after costal cartilage harvest, irrespective of the specific reconstructive procedure involved.

## Discussion

**Fig. 7:**
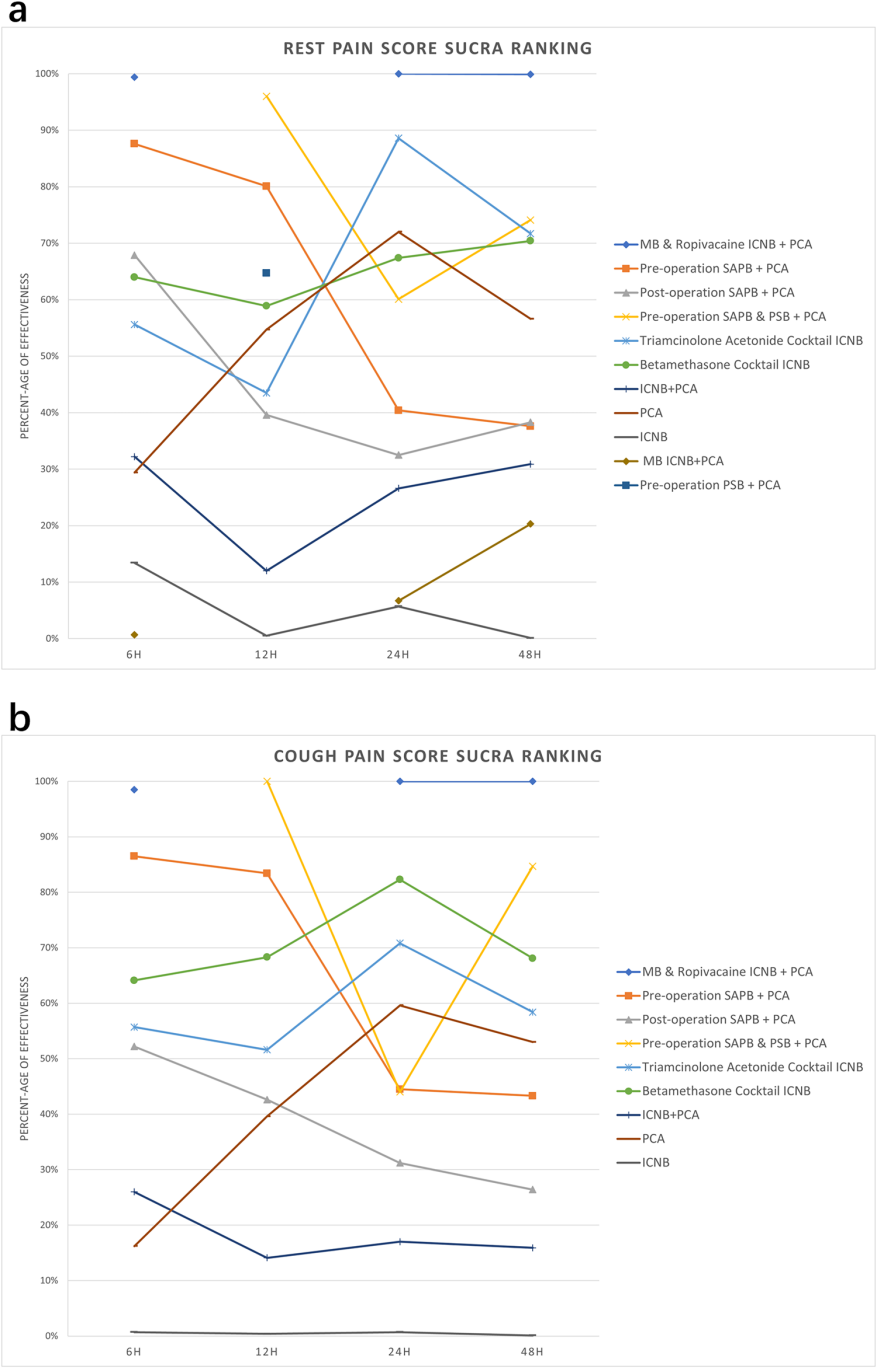
Pain scores SUCRA percentage. **a**. Resting pain scores SUCRA percentage at 6 h, 12 h, 24 h and 48 h postoperative. **b**. Coughing pain scores SUCRA percentage at 6 h, 12 h, 24 h and 48 h postoperative. *Note* Most interventions with additional measures to PCA showed reduced effectiveness between 12 and 48 h compared to the PCA-only group. This reduction in effectiveness is discussed in the manuscript and is attributed to the pharmacokinetic properties of local anesthetics, tachyphylaxis, and the evolving local inflammatory response.

This network meta-analysis has conducted a thorough evaluation of pain management methods in procedures involving the harvest of costal cartilage. It has yielded noteworthy results about the effectiveness of different pain-relieving treatments. After analyzing multiple studies that compared different interventions, we found that the “MB & Ropivacaine ICNB + PCA” strategy was the most beneficial in lowering postoperative resting and cough pain after 6, 24, and 48 h. Additionally, the “Pre-operation SAPB & PSB + PCA” intervention was identified as effective; however, it should be noted that the absence of data at the 12-h postoperative mark for the “MB & Ropivacaine ICNB + PCA” group limits the conclusiveness of this finding. The comparison between “SAPB & PSB + PCA’ and ‘MB & Ropivacaine ICNB + PCA” at 12 h remains incomplete, suggesting that further studies are needed to definitively determine the most effective intervention at this time point (Fig. 7a, b). These interventions notably outperformed others in SUCRA rankings, demonstrating superior pain control especially noted in early postoperative periods. Furthermore, “Pre-operation SAPB & PSB + PCA” also significantly decreased the need for rescue analgesia and effectively managed opioid-related adverse effects, enhancing patient safety and recovery.

However, it is important to note that most interventions with additional measures to PCA showed reduced effectiveness between 12 and 48 h compared to the PCA-only group. This reduction in effectiveness can be attributed to several factors. Firstly, the pharmacokinetic properties of local anesthetics such as ropivacaine, whose duration of action diminishes over time, play a significant role. Secondly, repeated exposure to these agents can lead to tachyphylaxis, reducing their efficacy. Thirdly, the local inflammatory response to surgical trauma evolves over time, potentially diminishing the localized effects of nerve blocks while systemic analgesia from PCA continues to exert a more consistent effect [30]. The discrepancy between the efficacy of ‘MB & Ropivacaine ICNB + PCA’ for primary outcomes and ‘Pre-operation SAPB & PSB + PCA’ for secondary outcomes can also be attributed to these factors. While ‘MB & Ropivacaine ICNB + PCA’ provides superior pain control immediately postoperatively, its effectiveness may not directly translate to secondary outcomes if the studies included in the analysis did not uniformly measure or report these outcomes for this specific intervention. These factors highlight the need for further research to explore strategies for sustaining the effectiveness of multimodal analgesic approaches beyond the immediate postoperative period.

The main results of our study are consistent with previous research that emphasizes the advantages of combining local anesthetics with anti-inflammatory drugs to extend the pain-relieving effects and reduce the adverse effects on the body as a whole [31]. A closer look at these multimodal strategies shows that their main benefits probably come from the way that local anesthetics and adjuvants improve the pharmacological profiles of each other [32]. Likely stem from synergistic mechanisms where local anesthetics and adjuvants enhance each other’s pharmacological profiles [32]. Ropivacaine, a local anesthetic, effectively blocks nerves, reducing immediate pain perception. Methylene blue and corticosteroids have anti-inflammatory properties that help alleviate local tissue reactions and prolong the duration of pain alleviation [33]. In addition, the utilization of methylene blue has been linked to a decrease in the overall toxicity of local anesthetics, hence improving the safety of these combinations [34]. The discrepancy observed between primary and secondary outcomes can also be explained by the pharmacokinetic profiles and mechanisms of action of these drugs. ‘MB & Ropivacaine ICNB + PCA’ is particularly effective in immediate pain control, whereas ‘Pre-operation SAPB & PSB + PCA’ reduces systemic inflammatory responses and the need for rescue analgesia.

A serratus anterior plane block before surgery and patient-controlled analgesia, with or without an enhanced parasternal block, worked very well together in the first 24 h after surgery, especially at 6 and 12 h. This effectiveness was observed in both resting and coughing conditions, resulting in high SUCRA rankings. However, the efficacy of this strategy diminished significantly in the later postoperative periods (at 24 and 48 h), where MB & Ropivacaine ICNB + PCA consistently ranked as the most effective approach, maintaining its superiority over all other pain management strategies according to league tables (Fig. 7a, 7b). This observed disparity in the performance of Pre-op SAPB + PCA(±PSB) and MB & Ropivacaine ICNB + PCA across different time points aligns with previous research findings that suggest varying duration effects of analgesic interventions [35, 36]. Literature indicates that SAPB, while effective initially, may not provide sustained analgesia beyond the early postoperative period, potentially due to the pharmacokinetics of the local anesthetics used [37], which tend to wear off without extended-release formulations or additional interventions [38]. Conversely, the sustained efficacy of MB & Ropivacaine ICNB + PCA could be attributed to the prolonged action of ropivacaine when used in combination with methylene blue, which may enhance the local anesthetic’s absorption and effect duration through its vasoconstrictive properties [39].

The data clearly indicates the drawbacks of using a single drug in typical intravenous continuous nerve block (ICNB) for managing postoperative pain. The study consistently shows low SUCRA ratings at all time periods, regardless of whether the patients were at rest or coughing. These findings are consistent with previous research that also indicates that using a single drug ICNB is not effective enough in relieving substantial postoperative pain in different surgical situations [40, 41]. The underperformance of traditional ICNB can be attributed to several pharmacological and physiological factors. Primarily, the duration of analgesia provided by traditional local anesthetics used in ICNB is often too short to cover the prolonged pain phases after surgeries involving costal cartilage harvest [4, 11]. Additionally, ICNB targets only the somatic nerves and does not address visceral pain, which can be significant in thoracic procedures [42, 43]. The localized effect of ICNB also limits its effectiveness in controlling diffuse or bilateral pain, common in extensive surgical recoveries. Moreover, traditional ICNB techniques often fail to achieve adequate analgesic coverage due to the variability in nerve location and the challenge of ensuring complete nerve blockade [44]. This often results in incomplete pain relief, necessitating supplemental systemic analgesics, which can introduce additional side effects. These shortcomings underscore the need for integrating multimodal pain management strategies that combine local anesthetics with other pharmaceutical agents or interventional techniques to enhance efficacy and patient outcomes.

However, it is essential to discuss the limitations of using non-autologous rib grafts as compared to autologous rib grafts, which are considered the gold standard. Autologous rib grafts, harvested from the patient’s own body, offer superior biocompatibility and lower risk of rejection or infection compared to non-autologous grafts [45]. Non-autologous rib grafts, whether from cadaveric sources (irradiated or fresh frozen) or synthetic materials, may introduce immunogenic responses leading to graft rejection or delayed healing [46]. Additionally, the structural integrity and mechanical properties of non-autologous grafts may not match those of autologous rib grafts, potentially resulting in compromised surgical outcomes [47]. Furthermore, alternative autologous sources such as ear or septal cartilage, while viable, may provide insufficient material for extensive reconstructive procedures and may also pose challenges in terms of graft viability and long-term stability. The use of irradiated or fresh frozen allografts, although advantageous in avoiding donor site morbidity, carries risks associated with disease transmission, immunogenic reactions, and potential for reduced biomechanical strength [48]. These factors highlight the importance of careful consideration when selecting graft materials and underscore the ongoing need for advancements in graft technology and immunomodulatory strategies to enhance the outcomes of reconstructive surgeries.

The combination of local anesthetics with other medicines or interventional procedures not only successfully relieves pain after surgery, but also greatly minimizes the need for additional pain relief medication and the occurrence of problems connected to opioid use. The dual advantage of this is essential for enhancing overall patient results, as decreasing dependence on rescue analgesia and opioids directly leads to a decrease in negative effects, a lower chance of opioid addiction, and potentially a faster recovery [49–51]. The reduction in rescue analgesia and opioid adverse effects is particularly important in the current healthcare environment, where there is a strong emphasis on minimizing opioid use due to the risks of addiction and the adverse side effects associated with these drugs [52]. By providing more effective pain control from the outset, these combined strategies decrease the likelihood that patients will experience pain severe enough to require additional opioid interventions. This approach not only improves patient comfort but also aligns with broader public health goals aimed at combating the opioid crisis.

This systematic review and network meta-analysis possess numerous strengths. Our study was comprehensive, including a wide range of analgesic therapies from multiple databases, which guarantees a strong synthesis of all the available evidence. Our wide range of knowledge enables us to thoroughly analyze and compare different pain management strategies, providing a distinct perspective on their effectiveness and results. Secondly, the use of advanced statistical tools like network meta-analysis enables us to draw indirect comparisons and provide a hierarchy of effective interventions, which is invaluable for clinical decision-making. However, our study is not without limitations. Despite our comprehensive search strategy, there is always the possibility of publication bias, as studies with positive outcomes are more likely to be published than those with negative or inconclusive results. Additionally, the heterogeneity in study designs, populations, and pain measurement scales across included studies may affect the generalizability of our findings. While we employed random effects models to account for this variability, the differences in surgical techniques, pain assessment tools, and patient demographics can still influence the outcomes. The majority of the include literature participants are Asian patients, more research on patients of different ethnicities is needed to obtain more comprehensive conclusions. Furthermore, most of the included studies were short-term, focusing on immediate postoperative outcomes; thus, long-term effects of these analgesic strategies remain less understood. Moreover, the specific details of analgesic administration, such as dosage and timing, were often poorly reported, limiting our ability to fully interpret the impact of these factors on pain management efficacy. Additionally, we conducted a sensitivity analysis to address the potential impact of including studies on rib harvest for rhinoplasty, which typically involves a smaller harvest size and lesser extent of dissection. The results indicated that the exclusion of rhinoplasty studies did not significantly alter the overall conclusions regarding the efficacy of pain management strategies. However, this approach underscores the inherent variability in surgical procedures and the need for more standardized reporting in future research. Despite these limitations, our findings consistently support the use of multimodal analgesic strategies for effective pain management following costal cartilage harvest. These strategies provide superior pain control and reduce the need for rescue analgesia and opioid-related adverse effects, aligning with current clinical priorities to enhance recovery and minimize opioid-related risks.

## Conclusion

This systematic review and network meta-analysis evaluated various advanced analgesic strategies for managing postoperative pain after costal cartilage harvest. Our analysis included 14 studies with 935 participants. We found that “MB & Ropivacaine ICNB + PCA” was most effective for primary pain outcomes, while “Pre-operation SAPB & PSB + PCA” showed superior results for secondary outcomes, likely due to differences in pharmacokinetic profiles and mechanisms of action. Both strategies significantly reduced pain scores and decreased the necessity for rescue analgesia and opioid-related adverse effects. These findings advocate for multimodal approaches integrating local anesthetics with other pharmacological or interventional techniques to enhance pain management efficacy and improve patient outcomes in reconstructive surgeries.

## Supplementary Information

Below is the link to the electronic supplementary material.

Supplementary file1 (DOCX 14 KB)
