# Supplementary material for: Comparative Efficacy of Postoperative Pain Management Techniques Following Costal Cartilage Harvest: A Systematic Review and Network Meta-analysis
Source: Aesthetic Plast Surg. 2024 Nov 11;49(3):929–49. doi: 10.1007/s00266-024-04430-2 (PMC11870948; doi:10.1007/s00266-024-04430-2)
Supplement: Supplementary file 1 — Supplementary file1 (DOCX 14 KB) [file 266_2024_4430_MOESM1_ESM.docx]

The Pubmed search strategy was as follows:

(((("Rhinoplasty"[MeSH Terms] OR "nasal augmentation"[Title/Abstract] OR "nose augmentation"[Title/Abstract]) OR ("Congenital Microtia"[MeSH Terms] OR "small ear"[Title/Abstract] OR "ear deformities"[Title/Abstract] OR "ear reconstruction"[Title/Abstract])) OR (Maxillofacial reconstruction)) OR ("Costal Cartilage"[MeSH Terms] OR "autologous rib cartilage"[Title/Abstract] OR "rib cartilage harvest"[Title/Abstract])) AND ("Anesthesia"[Mesh] OR "Analgesia"[MeSH Terms] OR "donor site analgesia"[Title/Abstract] OR "Pain"[MeSH Terms] OR "chest pain relief"[Title/Abstract] OR "postoperative pain control"[Title/Abstract])

The Cochrane search strategy was as follows:

Search Name:

Date Run: 16/03/2024 09:19:10

Comment:

ID Search Hits

#1 MeSH descriptor: [Rhinoplasty] explode all trees 416

#2 ("nasal augmentation" OR "nose augmentation"):ti,ab,kw (Word variations have been searched) 6

#3 #1 OR #2 421

#4 MeSH descriptor: [Congenital Microtia] explode all trees 18

#5 ("small ear" OR "ear deformities" OR "ear reconstruction"):ti,ab,kw (Word variations have been searched) 61

#6 #4 OR #5 77

#7 (“Maxillofacial reconstruction”):ti,ab,kw (Word variations have been searched) 19

#8 MeSH descriptor: [Costal Cartilage] explode all trees 14

#9 ("autologous rib cartilage" OR "rib cartilage harvest"):ti,ab,kw (Word variations have been searched) 7

#10 #8 OR #9 19

#11 MeSH descriptor: [Anesthesia] explode all trees 24909

#12 MeSH descriptor: [Analgesia] explode all trees 10561

#13 MeSH descriptor: [Pain] explode all trees 71541

#14 ("donor site analgesia" OR "chest pain relief" OR "postoperative pain control"):ti,ab,kw (Word variations have been searched) 2315

#15 #11 OR #12 OR #13 OR #14 92905

#16 #3 OR #6 OR #7 OR #10 521

#17 #15 AND #16 103

The Embase search strategy was as follows:

SourcesEmbase, MEDLINE, Preprints

Query('rhinoplasty'/exp OR 'nasal augmentation':ti,ab,kw OR 'nose augmentation':ti,ab,kw OR 'microtia'/exp OR 'small ear':ti,ab,kw OR 'ear deformities':ti,ab,kw OR 'ear reconstruction':ti,ab,kw OR 'maxillofacial reconstruction':ti,ab,kw OR 'rib cartilage'/exp OR 'autologous rib cartilage':ti,ab,kw OR 'rib cartilage harvest':ti,ab,kw) AND ('anesthesia'/exp OR 'analgesia'/exp OR 'donor site analgesia':ti,ab,kw OR 'chest pain relief':ti,ab,kw OR 'postoperative pain control':ti,ab,kw)

Mapped termsn/a
